# The Oxytocin Receptor in Spermatozoa May Originate From Both Spermatogenesis and Epididymal Maturation, and Regulates Capacitation

**DOI:** 10.1111/andr.70123

**Published:** 2025-09-27

**Authors:** Jesús Martínez‐Hernández, Ferran Garriga, Adeel Ahmad, Lorena Padilla, Carolina Maside, Sergi Bonet, Isabel Barranco, Jordi Roca, Luis Miguel Pastor, Marc Yeste

**Affiliations:** ^1^ Biotechnology of Animal and Human Reproduction (TechnoSperm), Institute of Food and Agricultural Technology University of Girona Girona Spain; ^2^ Unit of Cell Biology, Department of Biology, Faculty of Sciences University of Girona Girona Spain; ^3^ Department of Cell Biology and Histology, Faculty of Medicine, International Excellence Campus for Higher Education and Research “Campus Mare Nostrum”, IMIB‐Pascual Parrilla University of Murcia Murcia Spain; ^4^ Department of Medicine and Animal Surgery, Faculty of Veterinary Medicine, International Excellence Campus for Higher Education and Research “Campus Mare Nostrum”, IMIB‐Pascual Parrilla University of Murcia Murcia Spain

**Keywords:** apical blebs, epididymosomes, in vitro capacitation, oxytocin receptor, pig, spermatozoa

## Abstract

**Background:**

The oxytocin receptor (OR) is a G‐protein‐coupled receptor recently identified in human spermatozoa, whose origin and role in sperm physiology remain unknown.

**Objectives:**

In this study, using the pig as a model, we examine the presence of the OR in ejaculated spermatozoa through immunofluorescence and immunoblotting, and investigate the receptor's origin in the male gamete via immunohistochemistry in testicular and epididymal tissues. Additionally, we assess the involvement of the OR in in vitro capacitation and the acrosome reaction by utilizing physiological concentrations of agonists (oxytocin and carbetocin) and an antagonist (L‐371,257).

**Results:**

The results indicate that, in addition to the expected presence in ejaculated spermatozoa, the OR is expressed during spermatogenesis. Besides, this receptor is found in Leydig and Sertoli cells, as well as in the principal, basal, and apical cells of the epididymis. Furthermore, our data suggest that, during epididymal maturation, the OR could be incorporated in spermatozoa via extracellular vesicles within the apical blebs. The OR is involved in sperm capacitation, as the combination of the antagonist (L‐371,257) and the agonist (carbetocin) increases intracellular calcium levels and membrane lipid disorders, which are known as capacitation markers.

**Conclusions:**

The presence of the OR in mammalian spermatozoa could originate from both spermatogenesis and epididymal maturation. Moreover, in the male gamete, this receptor regulates sperm capacitation by interacting with its ligand in the female reproductive tract.

## 1 INTRODUCTION

The oxytocin receptor (OR) belongs to the rhodopsin‐like G‐protein‐coupled receptor (GPCR) family (class A) and is closely related to vasopressin receptors (VRs; V1aR, V1bR, and V2R)^1^, ^2^, ^3^. The OR is poorly selective and interacts with oxytocin and vasopressin with varying degrees of affinity,^4^, ^5^ as also do the different types of VRs (V1aR, V1bR, and V2R). This is because: (1) both the oxytocin receptor and VRs share high‐sequence homology, and (2) their ligands—oxytocin and vasopressin—are structurally related nonapeptides that only differ in two amino acids.^6^ The OR couples a trimeric complex of G proteins, consisting of a G_α_ subunit and a G_β/γ_ subunit. As the G_α_ subunit can be G_q/a11_, G_o_, or G_i_, this receptor can be involved in a plethora of intracellular signaling pathways.^2^

In myometrial smooth muscle cells, the activation of the OR is associated with an increase in intracellular calcium (Ca^2+^), thus modulating uterine contractions. This increase in Ca^2+^ is triggered by a G_αq/11_ protein coupled to the OR, which activates a phospholipase C (β) that causes the hydrolysis of phosphatidylinositol‐4,5‐bisphosphate (PIP_2_) to inositol‐1,4,5‐trisphosphate (IP_3_) and diacylglycerol (DAG). The IP_3_ stimulates the release of Ca^2+^ from the endo/sarcoplasmic reticulum, which, together with DAG, activates protein kinase C and evokes myometrium contractions via stimulation of Ca‐dependent calmodulin.^2^, ^7^ In myometrial cells, not only can the OR interact with G_q/11_ but also with G_i1_, G_i2_, and G_i3_, inhibiting the transmembrane adenylyl cyclase (mACY) and, therefore, the protein kinase A (PKA) pathway.^8^, ^9^

The hormone oxytocin is secreted by the female and male genital tracts. In humans and other mammalian species, it has been detected in the follicular fluid (in the order of picograms/mL)^10^, ^11^, ^12^, ^13^ and the oviduct.^14^, ^15^ In the male genital tract, local oxytocin synthesis has been described in the testis (Sertoli and Leydig cells),^16^, ^17^, ^18^, ^19^, ^20^ the epithelial cells of the epididymis,^18^, ^20^, ^21^ and the epithelial cells of the prostate.^22^ In addition to this, oxytocin has been detected and quantified in the seminal plasma (in the order of picograms/mL) of humans,^23^, ^24^ pigs^25^ (in order nanograms/mL), and horses.^20^

While the OR has been identified in human spermatozoa, its possible function is yet to be elucidated.^26^ Spermatozoa undergo a series of spatially and temporally ordered physiological changes within the female genital tract that prepare them to interact with the zona pellucida and fertilize the oocyte.^27^, ^28^ This process is known as capacitation and involves a series of molecular changes classified as early and late events.^29^ Early events begin when spermatozoa are exposed to high levels of bicarbonate and Ca^2+^ after their deposition in the female environment.^30^ The increase in pH activates soluble adenylyl cyclase (sACY), which stimulates PKA and phosphorylates its target proteins.^31^ The PKA phosphorylates sperm‐specific Ca^2+^ (CatSper) channels, thereby increasing the intracellular concentration of Ca^2+^, which favors membrane depolarization and activates sperm motility.^29^ This is followed by the start of late capacitation events, which involve the remodeling of the sperm plasma membrane. This membrane remodeling relies on the activation of phospholipid scramblases, which increase membrane fluidity and promote the efflux of cholesterol that is facilitated by acceptor proteins such as albumin.^29^, ^32^ Also, during late events, the plasma membrane hyperpolarizes and the motility pattern switches to hyperactivation. The progesterone present in the oviductal environment binds to its receptor on the sperm surface and triggers the acrosome reaction through an increment of intracellular Ca^2+^ via a T‐type voltage‐dependent Ca^2+^ channel.^29^, ^33^ During acrosomal exocytosis, hydrolytic enzymes are released, facilitating the passage of spermatozoa through cumulus cells and the zona pellucida. A phospholipase Cβ present in spermatozoa is involved in this process. As described above, this phospholipase hydrolyses PIP_2_ into IP_3_ and DAG. The IP_3_ binds the IP_3_ receptor (IP_3_R)‐gated calcium channel localized in the acrosome membrane, which then activates the Ca^2+^ release from the acrosome.^34^

Because of the presence of the OR in human spermatozoa and that of the natural ligand in the seminal plasma and female genital tract (follicular fluid and oviduct), we hypothesized that the OR could be involved in sperm capacitation. For this reason, and using the pig as a model, we interrogated the origin (spermatogenesis, epididymal maturation) and presence of the OR in mammalian spermatozoa by immunoblotting, immunohistochemistry (IHC), and immunofluorescence, and we addressed the involvement of this receptor in sperm capacitation and acrosomal reaction using different agonists and antagonists at physiological concentrations.

## 2 MATERIALS AND METHODS

### 2.1 Reagents

All chemicals were provided by Sigma‐Aldrich (Merck KGaA, Darmstadt, Germany) unless indicated otherwise.

### 2.2 Semen Samples

Seminal samples were purchased from a local farm (Gepork S.A.; Les Masies de Roda, Spain), operating under standard commercial conditions. Animals were sexually mature (between 18 and 24 months of age), housed under standard temperature and humidity conditions, fed a standard diet, and provided with water ad libitum. Handling of boars by the farm's staff followed the guidelines for animal welfare established by the Regional Government of Catalonia (Spain). As the authors did not manipulate any animal and the seminal doses involved in the study were intended for artificial insemination, no specific approval from an ethics committee was needed.

Males were collected through the gloved‐hand technique. Briefly, the sperm‐rich fraction of each ejaculate was immediately filtered through a gauze to remove the gel, and diluted 1:1 (v:v) in a long‐term extender (Vitasem, Magapor S.L., Zaragoza, Spain) at 37°C inside a collecting recipient. Commercial doses were obtained after further dilution and packaging into 90‐mL recipients at a concentration of 33 × 10^6^ spermatozoa/mL. Seminal doses were then cooled to 17°C and transported to our laboratory in a heat‐insulating container at 17°C. Once in the laboratory, sperm quality was assessed to ensure that all seminal doses fulfilled the minimum quality standards (viable spermatozoa ≥ 80%; total motile spermatozoa ≥ 70%; and morphologically normal spermatozoa ≥ 85%).

### 2.3 Tissue Samples

Testes and epididymides were obtained from adult boars that were culled for genetic replacement reasons from an AI center (Topigs Norsvin, Calasparra, Murcia), which complies with Spanish (ES300130640127; August 2006) and European (ES13RS04P; July 2012) regulations. These animals were healthy, fertile, and reproductively active. After slaughtering, tissue extracts (1 × 1 cm and 1 mm thick) from the medial testis, and from the caput, corpus, and cauda epididymis were collected and fixed in Bouin's solution at room temperature for 12 h. Following this, the tissue pieces were immersed in 70% alcohol, dehydrated, immersed in toluene, and embedded in paraffin. Slices of 4 µm thick were cut and mounted on glass slides for IHC and immunofluorescence of tissue samples.

### 2.4 Immunofluorescence of Sperm Samples

Three replicates were examined, each consisting of a pool of three seminal doses. Each sample was centrifuged at 600 × *g* for 5 min to remove the extender; the resulting pellet was resuspended in the same volume of phosphate‐buffered saline (PBS). Samples were again centrifuged at 600 × *g* for 5 min, and pellets were fixed in a 4% paraformaldehyde fixation solution (Thermofisher, Kandel, Germany) at room temperature for 20 min. Fixed samples were again centrifuged under the conditions mentioned before, and the resulting sperm pellets were resuspended in PBS to a final concentration of 5 × 10^6^ spermatozoa/mL. Fifty microliters were smeared on slides. Smears were permeabilized with PBS containing 1% Triton X‐100 at room temperature for 30 min, washed in PBS (5 min), and blocked with 0.02 M glycine in PBS at room temperature for 20 min in a humidity chamber. After that, samples were washed in PBS (5 min) and incubated with a polyclonal rabbit antibody against the OR (Proteitech, 23045‐1‐AP, Manchester, United Kingdom) at a dilution of 1/200 (v/v) in PBS containing 1% bovine serum albumin (BSA), at 4°C overnight in a humidity chamber. The next day, smears were washed in PBS (5 min) and incubated with a secondary donkey anti‐rabbit antibody conjugated with Alexa Fluor 488 (Invitrogen, A32731, Oregon, United States) at a dilution of 1/400 (v/v) in PBS containing 1% BSA at room temperature for 45 min, in a humidity chamber and darkness. Finally, smears were washed five times in PBS and mounted with one drop of mounting medium (Dako, Santa Clara, CA, United States) containing DAPI. Samples were kept in the dark before examination under a confocal laser‐scanning microscope (CLSM, Nikon A1R; Nikon, Tokyo, Japan) at 60×. The antibody specificity (blocking peptide assay) was confirmed by pre‐adsorption with its blocking peptide (five times in excess with respect to the primary antibody) at room temperature for 1 h. Negative controls were performed omitting the primary antibody.

### 2.5 Immunoblotting

One milliliter of the pooled semen samples was centrifuged at 650 × *g* for 5 min. The supernatant was discarded, and the proteins from the sperm pellet were extracted through resuspension in a lysis buffer (PBS supplemented with 1% SDS, 1% protease inhibitor cocktail [Sigma, Saint Louis, MO, United States], 0.1 mM phenyl‐methane‐sulfonylfluoride [PMSF], and 700 mM sodium orthovanadate). After thoroughly mixing, samples were incubated on ice for 30 min with occasional agitation, before centrifugation at 14,000 × *g* and 4°C for 20 min. Total protein in the supernatants was quantified using a detergent‐compatible method with a commercial kit (Bio‐Rad Laboratories, Hercules, CA, United States).

After determining protein concentration, samples were mixed with loading buffer (Laemmli Buffer [Bio‐Rad Laboratories] supplemented with 10% [v/v] β‐mercaptoethanol), and incubated at 95°C for 5 min. Ten microgram of total protein was loaded per lane into a Mini‐PROTEAN TGX 4%–15% Bis–Tris SDS‐PAGE precast gel (Bio‐Rad Laboratories). In the first lane of the gel, 10 µL of a Precision Plus Protein All Blue Prestained Standard (Bio‐Rad Laboratories) was loaded, and electrophoresis was run at 180 V for 45 min. Following this, proteins were transferred to a PVDF membrane by a Trans‐Blot Turbo device (1 A and 25 V for 30 min; Bio‐Rad Laboratories). Once proteins were transferred, membranes were blocked with Tris‐buffered saline with 0.1% Tween 20 (TBS‐Tween‐20 [TBS‐T]) supplemented with 5% BSA for 1 h in agitation. Membranes were incubated at 4°C overnight with the same primary antibody against the OR (Proteintech, 23045‐1‐AP, Manchester, United Kingdom) at a dilution of 1/1000 (v/v) in TBS‐T supplemented with 5% BSA. Membranes were subsequently washed three times in TBS‐T, and then incubated with a secondary HRP‐coupled goat anti‐rabbit antibody (Dako, Glostrup, Denmark) at a dilution of 1/2000 (v/v) in TBS‐T containing 5% BSA for 60 min at room temperature. Finally, membranes were washed five times, revealed with a chemiluminescent substrate (Millipore, Burlington, MA, United States), and visualized with a G:BOX Chemi XL system (SynGene, Frederick, MD, United States). As in the case of immunofluorescence, a blocking peptide assay (the peptide was five times in excess with respect to the primary antibody) and a negative control (no primary antibody) were included to confirm the specificity of the antibody.

### 2.6 Immunohistochemistry of Tissue Samples

Sections from the reproductive tissues were deparaffinized with xylene, and rehydrated in an increasing ethanol series (100%, 90%, and 70%) followed by washing in PBS. Endogen peroxidase activity was quenched with 0.3% H_2_O_2_ at room temperature for 30 min. Samples were blocked in PBS supplemented with 1% BSA at room temperature for 60 min in a humidity chamber. After that, slides were incubated with the same primary antibody against the OR (Proteintech, 23045‐1‐AP) at a dilution of 1/50 (v/v) in PBS containing 1% BSA at 4°C overnight in a humidity chamber. On the next day, slides were washed three times in PBS (5 min) and incubated with a secondary HRP‐coupled goat anti‐rabbit antibody (Dako, Glostrup, Denmark) at a dilution of 1/200 (v/v) in PBS containing 1% BSA at room temperature for 60 min, in a humidity chamber. Following this, tissue sections were incubated in a substrate‐chromogen solution containing 3,3′‐diaminobenzidine (SigmaFast tablets, Sigma) until a brown color corresponding to peroxidase activity was visible. Sections were subsequently counterstained with Harris acidified hematoxylin for 60 s. Finally, tissue sections were dehydrated in an increasing ethanol series (70%, 90%, and 100%), washed in xylene, and mounted with DPX medium (Scharlab, Barcelona, Spain). Tissue sections were examined under an Olympus BX‐51 bright‐field microscope (Olympus Co., Tokyo, Japan), and photographs were acquired with an Olympus DP‐25 digital camera connected to the microscope. The various cell types present in the epididymal epithelium were classified based on previous studies. Specifically, principal, clear, narrow, basal, and basophilic cells were classified following the indications provided by Briz et al.,^35^ whereas apical cells were identified following the criteria outlined by Ekstedt et al.^36^ As principal and clear cells are difficult to distinguish under bright‐field microscopy, and for the purpose of this study, both were considered as principal cells, taking into account that the principal cell becomes a clear cell after its apical cytoplasm is pinched into an apical protrusion.^35^

### 2.7 Immunofluorescence of Tissue Samples

Like in the previous section, tissue samples were deparaffinized and rehydrated before immunofluorescent analysis. Subsequently, samples were permeabilized with 1% Triton X‐100 in PBS for 30 min and blocked with 0.02 M glycine in PBS at room temperature for 20 min in a humidity chamber. Samples were incubated with three antibodies; a monoclonal mouse antibody against tetraspanin CD81 (Proteintech, 66866‐1, Manchester, United Kingdom) at a dilution of 1/50 (v/v), a polyclonal goat antibody against caveolin‐1 at a dilution of 1/100 (v/v) (Abcam, ab36152, Cambridge, United Kingdom), and a polyclonal rabbit antibody against OR at a dilution 1/50 (v/v) (Proteintech, 23045‐1‐AP), in PBS containing 1% BSA, at 4°C overnight in a humidity chamber. The next day, samples were washed three times in PBS (5 min each washing), and incubated with three secondary antibodies, donkey anti‐goat conjugated with Alexa fluor 647 (Thermo Fisher, A‐21447) at a dilution 1/400 (v/v), donkey anti‐mouse conjugated with Alexa fluor 405 (Abcam. ab175659) at a dilution 1/100 (v/v), and donkey anti‐rabbit conjugated with Alexa fluor 488 (Thermo Fisher, A‐21206) at a dilution 1/200 in PBS containing 1% BSA at room temperature for 45 min in a humid chamber. Slides were washed heavily (six times for 5 min) with PBS, and incubated with Sytox orange (Thermofisher, Oregon, United States) at a dilution of 1/5000 (v/v) at room temperature for 10 min to counterstain the nuclei. Finally, samples were washed and mounted with a fluorescent mounting medium (Dako, Santa Clara, CA, United States), and then examined under a CLSM (Nikon A1R; Nikon, Tokyo, Japan). The specificity of the three antibodies was tested by incubation with their blocking peptide (OR and CD81) or isotype control (caveolin‐1) at five times in excess. Negative controls (no primary antibody) were also included.

### 2.8 Transmission Electron Microscopy

Samples from each region of the epididymis (caput, corpus, and cauda) were rinsed in 0.16 M Sorensen phosphate buffer (pH 7.2) and prepared for transmission electron microscopy. Samples were initially fixed in 2.5% buffered glutaraldehyde and then post‐fixed in 1% osmium tetroxide. Samples were subsequently embedded in a Spurr ERL 402E resin. Following this, an Ultracut S ultramicrotome (Leica, Wetzlar, Germany) was employed to obtain ultrathin sections, which were subsequently mounted on copper grids, and contrasted with uranyl acetate and lead citrate. Grids were finally examined under a Hitachi I‐N 12A transmission electron microscope (Hitachi, Chiyoda‐ku, Tokyo).

### 2.9 In Vitro Capacitation

After confirming the presence of the OR in the sperm plasma membrane through immunofluorescence and immunoblotting, its physiological role during in vitro capacitation and the acrosome reaction was investigated by testing the effects of two agonists (oxytocin acetate salt, Bachem; and carbotecin, MedChemExpress) and one antagonist (L‐371,257; Tocris, Bristol, United Kingdom). Agonists were added to the media 15 min before the start of the experiment, and the antagonist (L‐371,257) was added to the medium 30 min before the start of the experiment to block the OR. The concentrations of agonists and the antagonist were established based on the literature,^25^, ^37^, ^38^ and were as follows: oxytocin acetate salt, 15 ng/mL; carbotecin, 200 nM; and L‐371,257, 50 nM.

For each independent experiment (*N* = 5), a pool of three different seminal doses was prepared. The pool was centrifuged at 600 × *g* and 17°C for 5 min, and sperm pellets were then resuspended in capacitating medium (20 mM HEPES, 96 mM NaCl, 4.7 mM KCl, 5.5 mM glucose, 21.6 mM sodium L‐lactate, 1 mM sodium pyruvate, 0.3 mM Na_2_HPO_4_, 0.4 mM MgSO_4_ × 7 H_2_O, 0.5 mM CaCl_2_ × 2 H_2_O, 3 mg/mL BSA, and 10 mM sodium bicarbonate) to a final concentration of 30 × 10^6^ spermatozoa/mL. After that, samples were split into six aliquots, which corresponded to the following treatments (control, oxytocin, carbotecin, L‐371,257, oxytocin + L‐371,257, and carbotecin + L‐371,257) containing the reagent concentrations indicated above.

Samples were incubated at 38.5°C, 100% humidity and 5% CO_2_ (Binder GmbH, Tuttlingen, Germany) for 180 min; in all samples, 10 µg/mL progesterone was added at 120 min. Analysis of sperm variables was conducted after 0, 60, 120, 130, and 180 min of incubation. At each relevant time point and for each treatment, sperm motility and kinematics were analyzed using a computer‐aided sperm analysis (CASA), and sperm viability, membrane lipid disorder, acrosome integrity, mitochondrial membrane potential (MMP), and intracellular levels of Ca^2+^, reactive oxygen species (ROS) and superoxide were determined by flow cytometry.

### 2.10 Evaluation of Sperm Motility

Sperm motility was evaluated using a CASA system consisting of a phase‐contrast microscope (Olympus BX41; Olympus, Tokyo, Japan) equipped with a warmed stage, a video camera, and the ISAS software (Integrated Sperm Analysis System V1.0; Proiser, S.L., Valencia, Spain). Three microliter of each sample was placed into a prewarmed (38°C) Leja chamber (Leja Products BV; Nieuw‐Vennep, The Netherlands) and observed under a negative phase‐contrast field. Images were captured and analyzed using the ISAS software, and each capture consisted of 25 pictures taken in 1 s. Two technical replicates of at least 1000 spermatozoa per replicate were analyzed per sample.

Percentages of total motile spermatozoa (progressive + non‐progressive) and progressively motile spermatozoa were determined. The different sperm kinematic parameters, including curvilinear velocity (VCL, µm/s), straight‐line velocity (VSL, µm/s), average path velocity (VAP, µm/s), amplitude of lateral head displacement (ALH, µm), beat cross frequency (BCF, Hz), linearity (LIN, %; LIN = VSL/VCL × 100), straightness (STR, %; STR = VSL/VAP × 100), and oscillation (WOB, %; WOB = VAP/VCL × 100) were also assessed. Spermatozoa were considered motile when their VAP was equal to or greater than 10 µm/s, and progressively motile when their STR was equal to or greater than 45%.

### 2.11 Flow Cytometry

A CytoFLEX device (Beckman Coulter, California, United States) was used to evaluate different sperm functional parameters during in vitro capacitation. The cytometer was equipped with forward scatter detector (FSD) and side scatter detector (SSD) that allowed for the discrimination of sperm cells from debris and cell aggregates. In the present study, the following parameters were assessed: sperm viability (SYBR‐14/propidium iodide [PI]), acrosome integrity (*Arachis hypogaea* (peanut) conjugated with FITC [PNA‐FITC]/[LIVE/DEAD] violet), membrane lipid disorder (merocyanine [M540]/Yo‐Pro‐1), intracellular superoxide levels (dihydroethidium [HE]/Yo‐Pro‐1), intracellular ROS levels (2′,7′‐dicholorodihydrofluorescin [H_2_DCFDA]/PI), intracellular Ca^2+^ levels (Fluo4‐AM/PI), and MMP (JC‐1/[LIVE/DEAD] far‐red). Samples were excited through a blue laser (488 nm) except LIVE/DEAD violet and LIVE/DEAD far‐red fluorochromes, which were excited with ultraviolet (405 nm) and red lasers (638 nm), respectively. The fluorescence emitted by LIVE/DEAD violet was detected with the PB450 channel (450/45). The fluorescence emitted by SYBR‐14, Yo‐Pro‐1, FITC‐PNA, JC‐1 monomers, Fluo4, and dicholorodihydrofluorescin (DCF^+^) was detected with the FITC channel (525/40); the fluorescence emitted by JC‐1 aggregates and ethidium (E^+^) was detected with the PE channel (585/42); the M540 fluorescence was detected with the ECD channel (610/20); the fluorescence from LIVE/DEAD far‐red was detected with the APC channel (660/20); and, finally, the fluorescence emitted by PI was collected through the PC5.5 channel (690/50). For each sample, two technical replicates of at least 5000 spermatozoa each were evaluated.

#### 2.11.1 Sperm Viability

Sperm viability was determined following the protocol described by Garner and Johnson.^39^ Briefly, samples were incubated with SYBR‐14 (final concentration: 7.6 µM) and PI (final concentration: 31.8 nM) at 38°C in the dark for 10 min. As a result of this staining, four subpopulations were identified: (i) viable green‐stained spermatozoa (SYBR‐14^+^/PI^−^); (ii) non‐viable red‐stained spermatozoa (SYBR14^−^/PI^+^); (iii) non‐viable (moribund) green‐ and red‐stained spermatozoa (SYBR14^+^/PI^+^); and (iv) debris particles (SYBR‐14^−^/PI^−^). Results are expressed as the recalculated percentage of viable spermatozoa after excluding the percentage of debris particles.

#### 2.11.2 Lipid Disorder Membrane

Lipid disorder of the sperm plasma membrane was evaluated following the protocol of Rathi et al.,^40^ as modified by Yeste et al.^41^ Spermatozoa were incubated with M540 (2.5 µM) and YO‐PRO‐1 (25 nM) at 38°C for 10 min. The basis of this protocol is that when the lipid disorder of the plasma membrane is high, M540 intercalates and emits red fluorescence. Four sperm subpopulations were identified: (1) viable spermatozoa with low membrane lipid disorder (M540^−^/Yo‐Pro‐1^−^), (2) viable spermatozoa with high membrane lipid disorder (M540^+^/Yo‐Pro‐1^−^), (3) non‐viable spermatozoa with low membrane lipid disorder (M540^−^/Yo‐Pro‐1^+^), and (4) non‐viable spermatozoa with high membrane lipid disorder (M540^+^/Yo‐Pro‐1^+^). Data from the double‐negative population were corrected using the percentages of debris particles in the SYBR14/PI staining, and the percentages of the other populations were recalculated accordingly. Results are expressed as the ratio of M540 intensity in total spermatozoa between the treatments and their respective control.

#### 2.11.3 Acrosome Integrity

Acrosome integrity was evaluated following the protocol Ritagliati et al.^42^ with minor modification. Two hundred microliter of each sperm sample was incubated with 1 µL LIVE/DEAD (LD) violet working solution (Thermo Fisher Scientific, Massachusetts, United States) at 38°C for 30 min in the dark, and then centrifuged at 650 × *g* for 5 min. Samples were subsequently resuspended in fixation solution (containing 4% paraformaldehyde, Thermofisher, Kandel, Germany; 0.1% Triton X‐100 and 0.3% Tween‐20) and incubated in a rotating shaker at room temperature for 30 min. Samples were centrifuged again at 800 × *g* for 5 min, and pellets were resuspended in 200 µL PBS. After analysis with flow cytometry, four sperm subpopulations were identified in dot‐plots: (1) viable spermatozoa with an intact acrosome (PNA‐FITC^+^/LD^−^), (2) viable spermatozoa with an exocytosed acrosome (PNA‐FITC^−^/LD^−^), (3) non‐viable spermatozoa with an intact acrosome (PNA‐FITC^+^/LD^+^), and (4) non‐viable spermatozoa with an exocytosed acrosome (PNA‐FITC^−^/LD^+^). Results are expressed as the percentage total spermatozoa with an exocytosed acrosome (PNA‐FITC^−^/LD^−^).

#### 2.11.4 Mitochondrial Membrane Potential

MMP was evaluated after staining with JC‐1 (final concentration: 750 nM) and the LIVE/DEAD fixable far‐red dead cell fluorochrome (Thermo Fisher Scientific, Massachusetts, United States), diluted at 1/8000 (v/v) in PBS, following the protocol of Garriga et al.^43^ Samples were then stained at 38°C in the dark for 30 min. High MMP results in JC‐1 aggregation (JC‐1_agg_) and the subsequent emission of orange fluorescence; in contrast, in sperm cells with low MMP, JC‐1 remains as a monomer (JC‐1_mon_) and emits green fluorescence. MMP was expressed as the ratio between the intensity of fluorescence of JC‐1_agg_ and the intensity of JC‐1_mon_ in viable spermatozoa.

#### 2.11.5 Intracellular Levels of Calcium

For the assessment of intracellular Ca^2+^ levels, double staining with Fluo4‐AM and PI was conducted. Fluo4‐AM penetrates the cell and emits fluorescence after de‐esterification and binding to Ca^2+^; thus, the greater the fluorescence of Fluo4, the greater the intracellular levels of Ca^2+^. In brief, samples were stained with Fluo4‐AM (final concentration: 1.17 µM) and PI (final concentration: 5.6 µM) at 38°C in the dark for 10 min. Data are expressed as the Fluo4 intensity in total spermatozoa in each treatment normalized to its respective control.

#### 2.11.6 Intracellular Levels of Superoxides

Intracellular levels of superoxide (O_2_^•−^) were evaluated through double‐staining with hydroethidine (HE, 5 µM) and YO‐PRO‐1 (31.25 nM).^44^ O_2_^•−^ oxidizes HE to ethidium (E), which emits red fluorescence. After staining, samples were incubated at 38°C in the dark for 20 min, and four subpopulations were identified in dot‐plots: (1) viable spermatozoa with low superoxide levels (E^−^/Yo‐Pro‐1^−^); (2) viable spermatozoa with high superoxide levels (E^+^/Yo‐Pro‐1^−^); (3) non‐viable spermatozoa with low superoxide levels (E^−^/Yo‐Pro‐1^+^); and (4) non‐viable spermatozoa with high superoxide levels (E^+^/Yo‐Pro‐1^+^). For each treatment, results are expressed as the intensity of E^+^ in total spermatozoa normalized to their respective control.

#### 2.11.7 Intracellular Levels of Reactive Oxygen Species

Intracellular levels of total ROS were determined through staining with 2′,7′‐dichlorodihydrofluorescein diacetate (H_2_DCFDA) and PI.^44^ ROS oxidize and de‐esterify H_2_DCFDA, which is not fluorescent, to DCF^+^, which, in contrast, emits green fluorescence. First, samples were incubated with H_2_DCFDA (final concentration at 50 µM) at 38°C in the dark for 20 min. Following this, PI was added to samples (final concentration: 6 µM), which were incubated under the same conditions for a further 5 min. For each treatment, total ROS levels are expressed as the intensity of DCF^+^ in total spermatozoa normalized to their respective control.

### 2.12 Statistical Analyses

A statistical package (spss Ver. 27.0 for Windows; IBM Corp., Armonk, NY, United States) was used for data analysis. Results were plotted with GraphPad Prism v.8 (GraphPad Software, La Jolla, CA, United States). First, data were tested for normal distribution (Shapiro–Wilk test) and homogeneity of variances (Levene test). The effects of treatments (oxytocin, carbotecin, L‐371,257, or their combination) on spermatozoa under in vitro capacitation conditions were evaluated through a linear mixed model, and pair‐wise comparisons were assessed by the post hoc Bonferroni test. In the linear mixed model, the different time points were the intra‐subjects factor, and the different treatments were the inter‐subjects factor.

Kinematics‐based sperm subpopulations were determined as described by Luna et al.^45^ Individual kinematic parameters were recorded for each sperm cell, and utilized as independent variables in a principal component analysis (PCA). The resulting matrix was rotated with the Varimax method, applying the Kaiser normalization. Regression scores obtained were subsequently used to perform a two‐step cluster analysis based on the log‐likelihood distance and Schwarz's Bayesian criterion. Thanks to this approach, three motile subpopulations were identified, and each spermatozoon was automatically assigned to one of these subpopulations. Subsequently, the percentages of spermatozoa belonging to each subpopulation were calculated for each treatment and time point, and compared through a linear mixed model as described above (inter‐subjects factor: treatment; intra‐subjects factor: time of storage).

In all statistical analyses, the level of significance was set at *p* ≤ 0.05. Data are represented as mean ± standard deviation (SD).

## 3 RESULTS

### 3.1 The Oxytocin Receptor Is Also Present in Pig Spermatozoa

**FIGURE 1:**
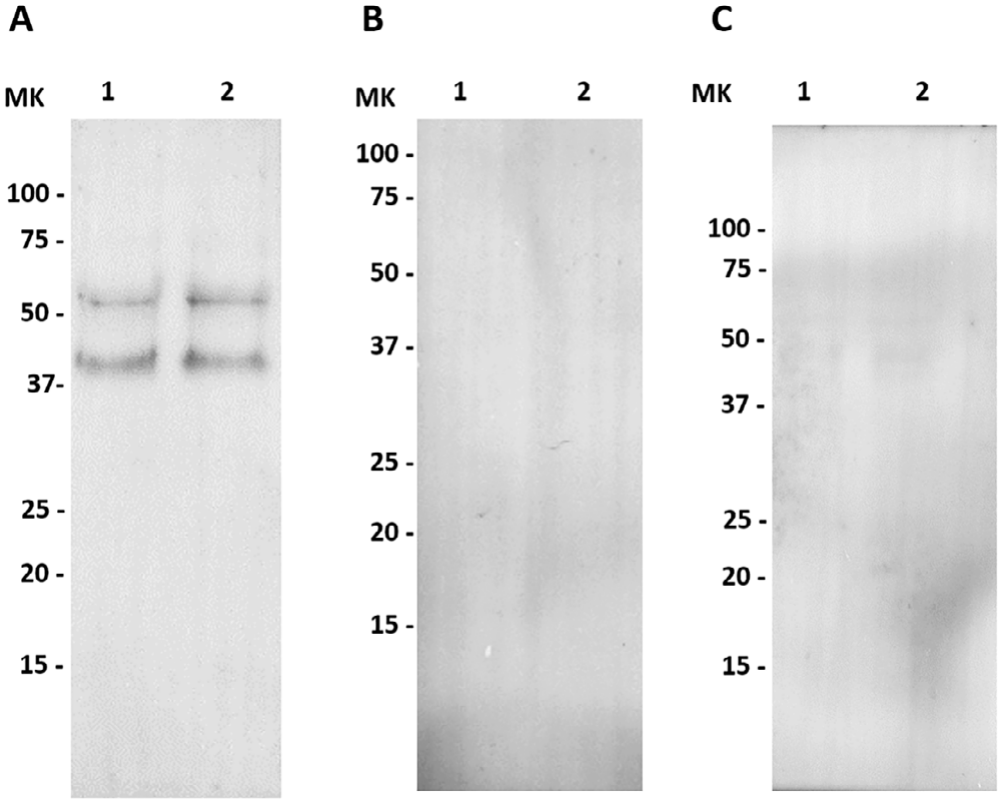
Immunoblotting of the oxytocin receptor (OR) in pig spermatozoa. (A) Incubation with the anti‐OR antibody revealed two bands (41 and 55 kDa); (B) peptide competition assay (blocking peptide) led the two signals to disappear, thus confirming the specificity of the primary antibody; (C) negative control (only secondary antibody), no signal detected. Lanes 1 and 2, corresponds to two pig sperm pools; MW, molecular weight.

**FIGURE 2:**
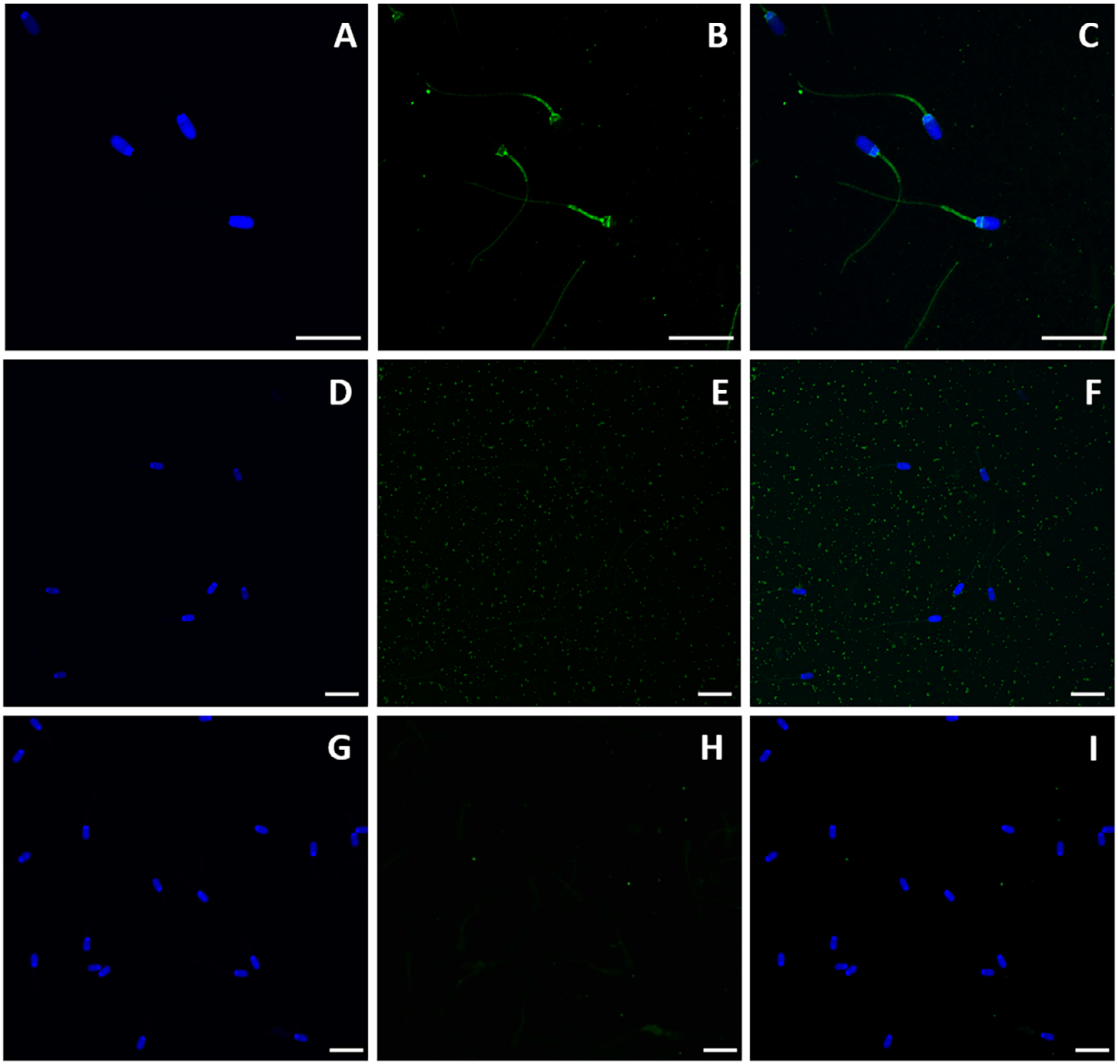
Immunostaining of the oxytocin receptor (OR) in pig spermatozoa. (A–C) Representative staining in spermatozoa. (D–F) Peptide competition assay of the sample (blocking peptide), no signal detected. (G–I) Negative control (only secondary antibody), no signal detected. The nucleus is shown in blue (DAPI), whereas the OR is shown in green. The bar corresponds 20  μ m.

The presence of the OR was confirmed by immunoblotting, as two specific bands of 41 and 55 kDa corresponding to this protein were observed in pig spermatozoa (Figure 1A). This observation was consistent with immunofluorescence staining, which allowed for the localization of the OR in porcine spermatozoa (Figure 2A). Specifically, by confocal microscopy, the OR was detected in the equatorial segment, post‐acrosomal region, connecting piece (neck) and midpiece. For both immunoblotting and immunofluorescence, blocking peptide assays and negative controls (without primary antibody) confirmed the specificity of the primary OR antibody (Proteintech, 23045‐1‐AP; Figures 1B,C and 2B,C).

### 3.2 The Oxytocin Receptor Is Also Present in Germ and Somatic Testicular Cells, and Epididymal Cells

**FIGURE 3:**
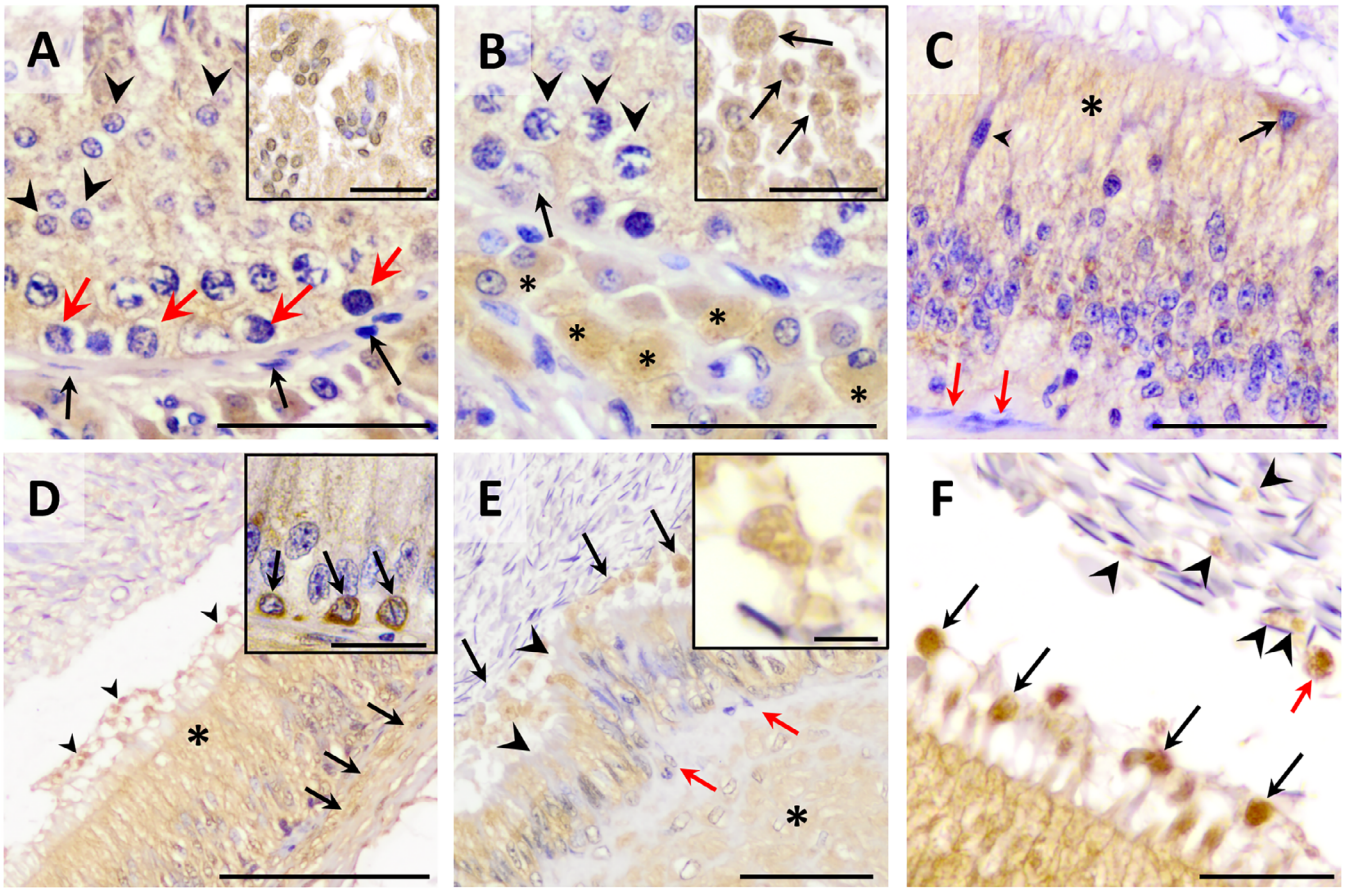
Immunohistochemistry of the oxytocin receptor (OR) in pig testis and epididymis. Testis (A, B). (A) Immunolabeling in the cytoplasm of round spermatids (arrowhead) and spermatogonium (red arrow). No staining was found in myoid cells (arrow). Insert detail: immunostaining in the cytoplasm of spermatids (in cap phase). (B) Positivity for the OR in the cytoplasm of spermatocytes (arrowhead), Sertoli (arrow) and Leydig cells (asterisk). Insert detail: residual bodies (arrow). Epididymis (C–F). (C) Caput epididymis. Positivity in the cytoplasm of principal (supranuclear region, asterisk) and apical cells (black arrow). No staining was observed in the narrow cell (arrowhead) or in the connective tissue underlying the epididymal epithelium (red arrow). (D) Corpus epididymis. Immunostaining of principal cells (asterisk), apical blebs (arrowhead), and smooth muscle layer (arrows). Insert detail: Positive basal cells (arrow). (E) Cauda epididymis. Apical blebs emerging from the principal cells are observed (arrows). Some principal cells do not show immunostaining (arrowhead). Beneath the epithelium, a layer of connective tissue (red arrow) negative for OR is observed, adjacent to the smooth muscle layer (asterisk), which shows positive OR expression. Insert: apical blebs in the epididymal lumen showing vesicular content inside. (F) Detail of apical blebs emerging from the principal cells in corpus region with a granular or vesicular into content (arrows). Apical blebs were observed in the epididymal lumen near spermatozoa (red arrow), along with other smaller vesicular structures (arrowhead), possibly derived from the disintegration of these apical blebs. Scale bars: (A) 50 µm; (B) 50 µm; (C) 50 µm; (D) 100 µm; (E) 20 µm, (F) 20 µm. Inserts details (A–D); 20 µm, E; 5 µm.

IHC for bright‐field microscopy revealed that the OR is expressed in the testis and epididymis. In the testis, the OR was found to be expressed in the interstitium, specifically in the membrane and cytoplasm of Leydig cells, and in the seminiferous tubules. In the latter case, whereas spermatogonia and spermatocytes showed weak labeling for the OR, spermatids, especially the late ones, and residual bodies showed intense staining; yet, not all spermatids were positive for the OR. In addition, the membrane and cytoplasm of Sertoli cells showed positive labeling for the OR, whereas this protein was absent from either the myoid cells of the seminiferous tubular wall or testicular spermatozoa (Figure 3A,B). In the epididymal regions (caput, corpus, and cauda), principal (or clear) and apical cells exhibited positive immunoreactivity. In contrast, narrow cells did not show any detectable immunopositivity, and only some basal cells in the caput and corpus displayed a positive signal. The connective tissue layer underlying the epididymal epithelium was also negative for the OR, and increased in thickness toward the cauda portion of the epididymis (Figure 3C–E). In contrast, epididymal smooth muscle cells were positive for OR immunostaining. Remarkably, a strongly positive structure for the OR, corresponding to apical blebs (especially from the corpus region), was observed to emerge from the principal epididymal cells. These vesicular structures, which displayed a heterogeneous and granular content positive for the OR, appeared to accumulate in the periphery of the epididymal lumen until they interacted with spermatozoa (Figure 3E,F). Finally, using bright‐field IHC, no OR positivity was observed in epididymal spermatozoa.

**FIGURE 4:**
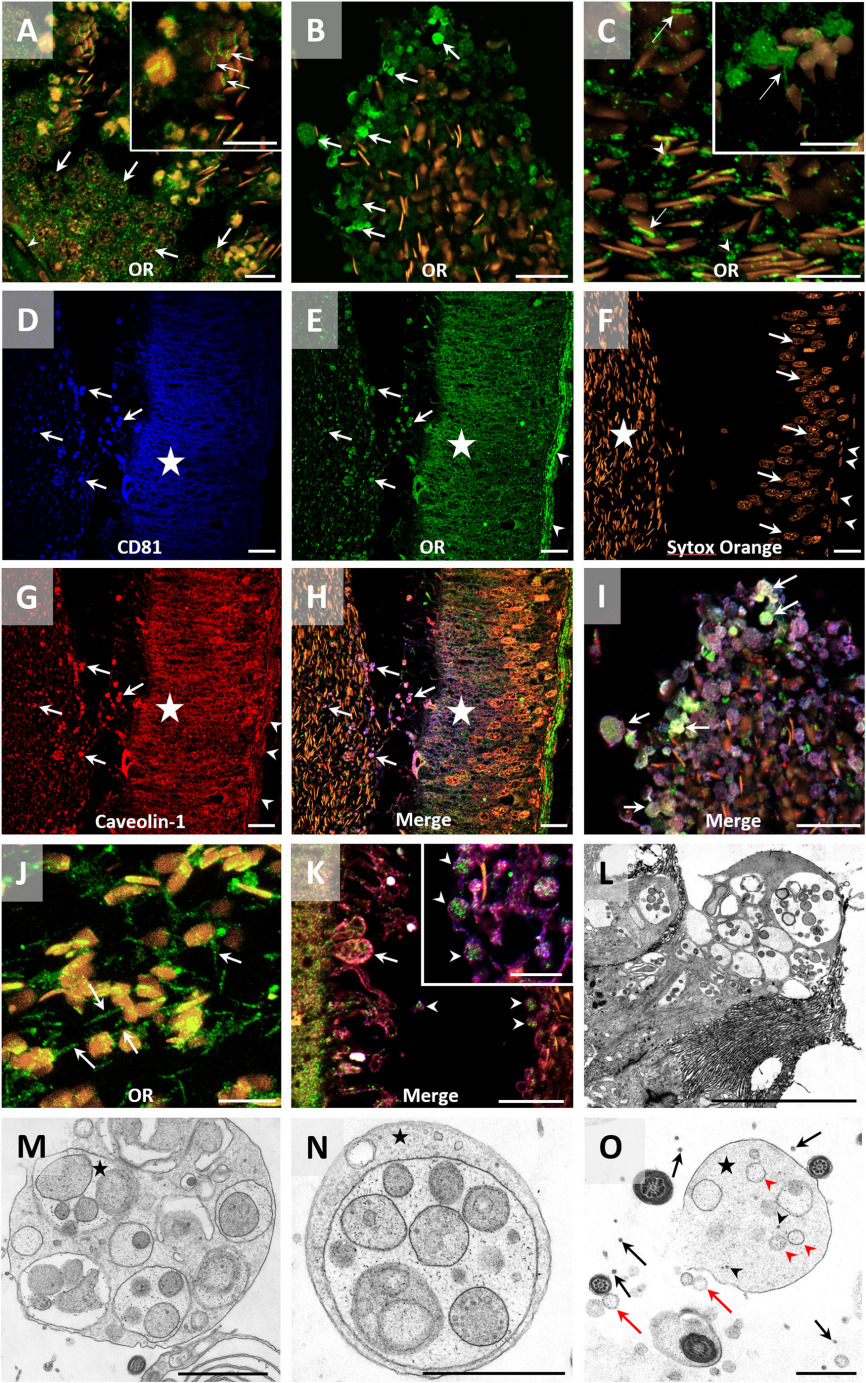
Confocal microscopy (immunofluorescence) for pig testis and epididymis, and transmission electron microscopy (TEM) for pig epididymis. (A) 3D projection (z‐stack) in testis. Immunolabeling for the oxytocin receptor (OR) was observed in germinal cells (arrow), whereas while myoid cells were negative (arrowhead). Insert detail: The OR was found in the post‐acrosomal region of pig spermatozoa in the lumen of seminiferous epithelium (arrow). (B) Spermatozoa from the corpus of the epididymis. Strong labeling for the OR was observed in the apical blebs, which showed vesicular heterogeneity in content and size (arrow). (C) 3D projection (z‐stack) of spermatozoa in the corpus epididymis. Labeling for the OR was observed in the post‐acrosomal region of spermatozoa (arrow) and probably in the disintegrated apical blebs in close contact with spermatozoa (arrowhead). Insert detail: Positivity for the OR in the sperm tail. (D–I). Colocalization of CD81, caveolin‐1, and the OR in the epididymal tissue. (D) The apical blebs (arrow) and supranuclear region of the epididymal principal cells (star) were positive for tetraspanin CD81. (E) OR positivity was observed in the apical blebs (arrow), principal cells (star) and smooth muscle cell layer (arrowhead). (F) Staining with SYTOX orange, which stains the nuclei of spermatozoa in the lumen (star), the nuclei of the cells in the epididymal epithelium (arrow) and the nuclei of smooth muscle cells in the muscular wall (arrowhead). (G) Caveolin‐1 immunostaining in apical blebs (arrow), cells of the epididymal epithelium (star), and muscle layer (arrowhead). (H) Merge of the above four channels, which showed the colocalization of the OR, CD81, and caveolin‐1 in the apical blebs (arrows) of the epididymal epithelium. The supranuclear region of the principal cells also depicted strong colocalization of these three antibodies (star). (I) Merge of the four channels of the image (B), showing in more detail the colocalization of CD81, cav‐1, and OR in the apical blebs and derived structures (arrows). (J) 3D projection (z‐stack) of spermatozoa from cauda epididymis. Immunolabeling for the OR is detected in the sperm tail (white arrow). (K) A merged four‐channel image shows an apical bleb emerging from the epididymal epithelium in the caput region (white arrow), along with free apical blebs in the lumen in proximity to spermatozoa (arrowhead). Insert detail: higher magnification of the free apical blebs (arrowhead) shows the presence of the OR within their lumen. (L–O) Transmission electron microscopy (TEM). A potential sequence of apical bleb disintegration into smaller vesicular substructures is shown. (L) An apical bleb emerging from an epididymal epithelial cell (corpus) containing heterogeneous multivesicular content. Note the parallelism with confocal microscopy images. (M) Free apical bleb containing heterogeneous substructures (star) in the epididymal lumen. (N) Putative free vesicular substructures resembling those found inside the apical bleb shown in M (star). (O) Smaller individualized vesicular structures, likely exosomes (arrow), exhibiting diverse morphologies and localized among or adjacent to spermatozoa. Free vesicular substructures (star) appear to release these exosomes (arrowhead). Other single extracellular vesicles larger (red arrow) than exosomes are also observed both in the epididymal lumen and within the substructures (red arrowhead) of the apical blebs (star). Scale bars: (A–C, I, J) 10 µm; (D–H, K) 20 µm; (L) 5 µm; (M, N) 2 µm; (O) 1 µm. Inserts detail 10 µm.

Immunofluorescence under a confocal microscope confirmed the presence of the OR in the Leydig, Sertoli, and germ cells of the testis. In addition, OR labeling was occasionally observed in the post‐acrosomal region of testicular spermatozoa (Figure 4A). In the epididymis, confocal microscopy also confirmed the same findings observed under bright‐field microscopy: the presence of the OR in the epithelium, apical blebs, and the smooth muscle cell layer. Furthermore, OR staining was observed in the post‐acrosomal region and in the tail of some epididymal sperm cells from different regions of the epididymis (Figure 4C,J). As in previous cases, the specificity of the primary antibody against the OR was confirmed by both blocking peptide assays and negative controls (see Supporting Information Figure 1).

### 3.3 The Presence of the OR in the Epididymal Apical Blebs Suggests That This Receptor Is Incorporated Into Spermatozoa During Epididymal Maturation

The presence of the OR in the apical blebs suggested its incorporation into spermatozoa during epididymal maturation. To interrogate this further, a triple immunostaining targeting two extracellular vesicle (EV) markers, CD81 and caveolin‐1,^46^ and the OR was performed using confocal microscopy. Interestingly, the epididymal apical blebs showed positive labeling for these markers, with the OR colocalizing with CD81 and caveolin‐1. Furthermore, these apical blebs were observed to accumulate at the periphery of spermatozoa in the epididymal lumen, remaining in close contact with them. In addition, and consistent with these data, the apical region of the epididymal epithelium was positive for the two EV markers (CD81 and caveolin‐1) and for the OR (Figure 4D–I). Apical blebs emerging from the epididymal epithelium were observed to contain the OR with a heterogeneous distribution. In some cases, the OR colocalized with the peripheral membrane of the apical blebs, whereas in others, it was detected within smaller substructures that appeared to be derived from the breakdown of these blebs (see detail in Figure 4K). Transmission electron microscopy confirmed that these apical blebs were released into the epididymal lumen and presented a heterogeneous content, as previously suggested by bright‐field and confocal microscopy analyses. Additionally, we were able to observe what appeared to be a pattern/sequence of disintegration/breakdown of these apical blebs into smaller subproducts within the epididymal lumen (Figure 4L–O). Finally, the specificity of the three antibodies was tested by incubation with their respective blocking peptide (OR and CD81) or isotype control (caveolin‐1), as well as through appropriate negative controls (see Supporting Information Figure 2).

### 3.4 The Activation of the OR Does Not Induce Changes in Sperm Motility

No changes in sperm motility variables were observed in any of the treatments tested (Table 1). The evaluation of motile sperm subpopulations started with a PCA analysis, which revealed that two principal components explained up to 81.46% of the variance. The first component was tightly related to VCL, VSL, VAP, ALH, and BCF, whereas the second component was linked to LIN, STR, and WOB (Table 2). Subsequently, a cluster analysis was performed using the regression scores from each of these two components. A total of three sperm subpopulations, exhibiting differences in their kinematics variables, were obtained. Table 3 describes the kinematic characteristics of spermatozoa belonging to each subpopulation. Subpopulation 1 (SP1) comprised the most linear and fastest spermatozoa and represented 49.15% of motile spermatozoa. Subpopulation 2 (SP2) was characterized by the slowest spermatozoa but with moderate linearity and corresponded to 30.69% of motile spermatozoa. Subpopulation 3 (SP3) contained the remaining 20.15% of motile spermatozoa and was characterized by low linear movement and moderate speed. Noticeably, blocking of the OR did not induce changes in the distribution of motile sperm subpopulations (*p *> 0.05).

**TABLE 1:** Sperm motility and kinematics in samples treated with oxytocin receptor agonists (oxytocin and carbotecin), antagonist (L‐371,257), and their combination (oxytocin + L‐371,257 and carbotecin + L‐371,257), under in vitro capacitation conditions.

	0	60	120	130	180
PROGRESSIVE MOTILITY (%)					
Control	51.4 ± 8.9	43.2 ± 9.0	39.2 ± 13.1	16.0 ± 11.3	10.4 ± 6.8
Oxytocin	56.3 ± 7.2	44.3 ± 12.1	38.2 ± 10.2	17.2 ± 12.8	11.9 ± 7.0
Carbotecin	49.2 ± 12.7	42.0 ± 19.1	30.9 ± 10.5	14.5 ± 9.0	11.3 ± 6.8
L‐371.257	58.7 ± 3.9	50.6 ± 12.6	38.5 ± 12.1	14.2 ±9.0	13.8 ± 10.3
Oxy + L371	57.1 ± 5.9	49.8 ± 11.6	35.8 ± 10.8	12.4 ± 7.1	12.2 ± 7.1
Carb + L371	53.7 ± 9.3	41.9 ± 10.5	35.7 ± 9.8	14.9 ±12.7	9.5 ± 6.8
TOTAL MOTILITY (%)					
Control	64.4 ± 11.0	54.7 ± 9.5	51.1 ± 14.3	26.7 ± 13.8	19.3 ± 11. 6
Oxytocin	70.4 ± 10.0	57.5 ± 15.5	49.5 ± 10.1	25.7 ± 14.2	19.4 ± 6.9
Carbotecin	62.9 ± 15.9	53.4 ± 21.2	42.7 ± 10.2	23.3 ± 11.4	19.1 ± 8.5
L‐371.257	73.5 ± 5.1	62.0 ± 13.3	51.9 ± 11.8	22.3 ± 10.7	22.3 ± 12.7
Oxy+L371	70.4 ± 7.3	61.6 ± 12.5	45.5 ± 9.9	23.2 ± 15.1	20.4 ± 9.9
Carb + L371	66.4 ± 12.7	52.6 ± 11.5	45.7 ± 8.9	25.9 ± 20.7	16.2 ± 6.8
VCL (µm/s)					
Control	85.5 ± 9.7	74.2 ± 9.4	71.8 ± 18.2	47.1 ± 10.0	42.4 ± 5.8
Oxytocin	89.3 ± 9.2	77.2 ± 15.1	73.1 ± 10.6	50.8 ± 10.8	42.7 ± 7.0
Carbotecin	86.9 ± 11.9	76.4 ± 19.8	66.1 ± 3.6	47. ± 8.0	41.3 ± 4.4
L‐371.257	92.8 ± 9.3	81.1 ±17.2	70.1 ± 10.2	55.5 ± 17.6	42.6 ± 6.4
Oxy + L371	91.5 ± 10.7	77.5 ± 12.5	71.5 ± 10.8	49.3 ± 4.7	44.2 ± 8.5
Carb + L371	87.5 ± 5.6	77.9 ± 12.0	71.6 ± 9.0	41.4 ± 12.9	43.9 ± 11.5
VSL (µm/s)					
Control	53.3 ± 6.9	52.4 ± 9.8	48.9 ± 16.1	19.3 ± 4.9	16.0 ± 4.9
Oxytocin	56.8 ± 4.9	55.1 ± 13.5	49.8 ± 11.0	19.5 ± 5.9	19.5 ± 5.9
Carbotecin	55.5 ± 8.9	55.4 ± 14.9	42.6 ± 7.4	17.8 ± 3.3	17.8 ± 3.3
L‐371.257	59.6 ± 4.9	57.9 ± 14.6	46.4 ± 10.9	24.9 ± 16.3	18.5 ± 6.5
Oxy + L371	60.9 ± 8.3	56.4 ± 13.0	45.7 ± 11.7	18.4 ± 5.2	18.6 ± 6.2
Carb + L371	58.1 ± 6.0	56.9 ± 11.2	46.2 ± 12.7	17.0 ± 7.0	17.7 ± 6.5
VAP (µm/s)					
Control	63.3 ± 9.7	56.4 ± 9.9	52.8 ± 17.1	25.0 ± 6.4	21.7 ± 4.6
Oxytocin	66.8 ± 8.0	59.9 ± 15.1	53.9 ± 10.9	27.4 ± 7.8	24.6 ± 5.1
Carbotecin	64.3 ± 12.1	59.3 ± 16.8	46.5 ± 6.8	25.1 ± 6.5	23.6 ± 3.2
L‐371.257	69.1 ± 10.2	62.2 ± 15.2	50.6 ± 10.5	30.7 ± 15.1	23.9 ± 5.7
Oxy + L371	69.6 ± 12.2	59.9 ± 13.6	50.0 ± 11.5	25.8 ± 4.2	24.1 ± 6.1
Carb + L371	66.1 ± 7.7	60.8 ± 11.8	50.6 ± 11.3	22.1 ± 7.7	24.0 ± 3.2
LIN (%)					
Control	62.4 ± 5.2	70.2 ± 5.1	67.2 ± 6.5	41.1 ± 10.5	37.3 ± 7.4
Oxytocin	63.8 ± 4.0	70.8 ± 4.4	67.4 ± 7.2	41.5 ± 9.0	45.6 ± 9.9
Carbotecin	63.1 ± 4.0	72.3 ± 3.0	64.2 ± 8.5	39.9 ± 9.0	43.0 ± 6.1
L‐371.257	64.0 ± 4.2	70.9 ± 7.2	65.5 ± 9.3	41.9 ± 14.0	42.8 ± 10.4
Oxy + L371	66.5 ± 2.6	72.1 ± 6.3	63.1 ± 7.1	37.2 ± 9.7	41.7 ± 9.2
Carb + L371	66.3 ± 2.7	72.7 ± 5.0	63.6 ± 11.1	40.4 ± 10.0	40.3 ± 8.9
STR (%)					
Control	84.7 ± 7.6	92.8 ± 2.0	92.5 ± 1.2	76.5 ± 7.7	72.8 ± 6.8
Oxytocin	81.2 ± 13.1	88.3 ± 9.0	88.0 ± 7.7	70.9 ± 11.3	71.4 ± 14.5
Carbotecin	81.7 ± 14.2	89.3 ± 8.7	85.9 ± 9.6	66.8 ± 13. 7	67.6 ± 15.8
L‐371.257	82.2 ± 12.2	89.4 ± 6.9	86.6 ± 8.6	68.9 ± 24.3	69.7 ± 11.9
Oxy + L371	84.2 ± 10.6	90.4 ± 7.6	84.7 ± 15.9	63.8 ± 11.1	69.3 ± 14.5
Carb + L371	84.2 ± 10.4	89.5 ± 8.2	86.1 ± 9.2	69.0 ± 9.5	65.4 ± 18.4
WOB (%)					
Control	72.3 ± 3.0	73.8 ± 4.6	69.7 ± 7.7	50.9 ± 8.5	50.2 ± 5.6
Oxytocin	73.4 ± 2.6	75.7 ± 5.0	70.6 ± 6.7	52.6 ± 7.2	54.8 ± 6.9
Carbotecin	71.0 ± 6.0	76.9 ± 3.4	66.7 ± 7.5	51.9 ± 7.3	56.2 ± 4.7
L‐371.257	72.6 ± 4.8	74.2 ± 7.5	69.1 ± 8.8	54.8 ± 11.3	53.3 ± 6.2
Oxy + L371	74.1 ± 6.2	74.6 ± 7.0	67.1 ± 6.1	49.7 ± 6.4	51.9 ± 7.0
Carb + L371	74.6 ± 5.7	76.3 ± 5.7	66.8 ± 9.8	52.0 ± 7.0	53.3 ± 6.8
ALH (µm)					
Control	2.9 ± 0.3	2.7 ± 0.2	2.9 ± 0.4	2.0 ± 0.8	2.5 ± 0.7
Oxytocin	3.1 ± 0.4	2.8 ± 0.4	2.8 ± 0.3	2.4 ± 0.3	2.2 ± 0.1
Carbotecin	3.1 ± 0.4	2.7 ± 0.5	2.6 ± 0.2	2.2 ± 0.3	2.0 ± 0.4
L‐371.257	3.3 ± 0.4	2.8 ± 0.5	2.8 ± 0.2	2.6 ± 0.4	2.2 ± 0.2
Oxy + L371	3.1 ± 0.2	2.7 ± 0.3	2.8 ± 0.2	2.3 ± 0.4	2.2 ± 0.3
Carb + L371	3.0 ± 0.2	2.7 ± 0.3	2.7 ± 0.1	2.0 ± 0.7	2.3 ± 0.4
BCF (Hz)					
Control	7.9 ± 0.7	8.3 ± 0.3	8.2 ± 0.2	6.2 ± 0.9	5.9 ± 1.2
Oxytocin	7.8 ± 0.8	8.0 ± 0.2	8.1 ± 0.6	6.6 ± 0.7	5.9 ± 1.1
Carbotecin	7.9 ± 1.0	8.5 ± 0.2	8.1 ± 0.4	5.9 ± 1.0	6.0 ± 0.6
L‐371.257	8.0 ± 0.8	8.4 ± 0.3	8.0 ± 0.4	6.2 ± 1.9	5.9 ± 1.2
Oxy + L371	8.0 ± 0.5	8.4 ± 0.4	8.2 ± 0.3	5.9 ± 1.1	5.4 ± 1.8
Carb + L371	8.2 ± 0.6	8.3 ± 0.3	8.3 ± 0.6	5.4 ± 2.2	4.8 ± 1.7

Abbreviations: ALH, amplitude of lateral head displacement; BCF, beat cross frequency; LIN, linearity; STR, straightness; VAP, average path velocity; VCL, curvilinear velocity; VSL, straight‐line velocity; WOB, oscillation index.

**TABLE 2:** Resulting PCA components based on kinematic characteristics evaluated by CASA.

Principal component	Variance (%)	Parameter	*a_ij_* ^2^
Component 1	56.08	VCL	0.978
		VSL	0.726
		VAP	0.842
		ALH	0.900
		BCF	0.522
Component 2	25.37	LIN	0.972
		STR	0.817
		WOB	0.851

Abbreviations: ALH, amplitude of lateral head displacement; BCF, beat cross frequency; LIN, linearity; STR, straightness; VAP, average path velocity; VCL, curvilinear velocity; VSL, straight‐line velocity; WOB, oscillation index.

The factor loading (*a_ij_* ^2^) represents the correlation between a given kinematic parameter and the corresponding principal component.

**TABLE 3:** Kinematic characteristics (mean, range) of the three sperm motile subpopulations (SP1, SP2, and SP3) identified in sperm samples with different treatments under in vitro capacitation conditions.

	SP1	SP2	SP3
*N*	32,641 (49.15%)	20,401 (30.69%)	13,369 (20.15%)
VCL (µm/s)	109.16 (46.76–223.42)	37.12 (10.00–108.43)	71.34 (10.32–231.18)
VSL (µm/s)	84.68 (35.13–199.66)	21.91 (2.57–68.34)	15.18 (0.00–61.95)
VAP (µm/s)	87.83 (39.19–194.28)	25.35 (3.06–83.68)	37.13 (2.97–157.94)
LIN (%)	78.08 (23.83–100)	59.83(18.15–100)	20.62 (0.00–52.65)
STR (%)	94.27 (31.84–100)	83.82 (34.78–100)	41.73 (0.00–100)
WOB (%)	80.99 (28.49–100)	70.41 (15.09–100)	52.32 (5.13–100)
ALH (µm)	3.26 (0.31–9.16)	1.64 (0.26–4.40)	3.07 (0.63–10.70)
BCF (Hz)	8.91 (0.00–20.00)	5.30 (0.00–18.00)	5.50 (0.00–18.00)
DANCE (µm^2^/s)	375.46 (19.66–1944.54)	71.59 (4. 36–382.43)	264.98 (7.87–2132.98)
MAD	−0.01 (−44.11 to 43.25)	−0.11 (−43.59 to 42.97)	0.06 (−37.07 to 41.68)

Abbreviations: ALH, amplitude of lateral head displacement; BCF, beat cross frequency; LIN, linearity; MAD, mean angular displacement (absolute values); STR, straightness; VAP, average path velocity; VCL, curvilinear velocity; VSL, straight‐line velocity; WOB, oscillation index.

### 3.5 The Activation of the OR During Sperm Capacitation Increases Intracellular Ca^2+^ Levels and Membrane Lipid Disorder, Without Affecting Sperm Viability or Acrosome Integrity

**FIGURE 5:**
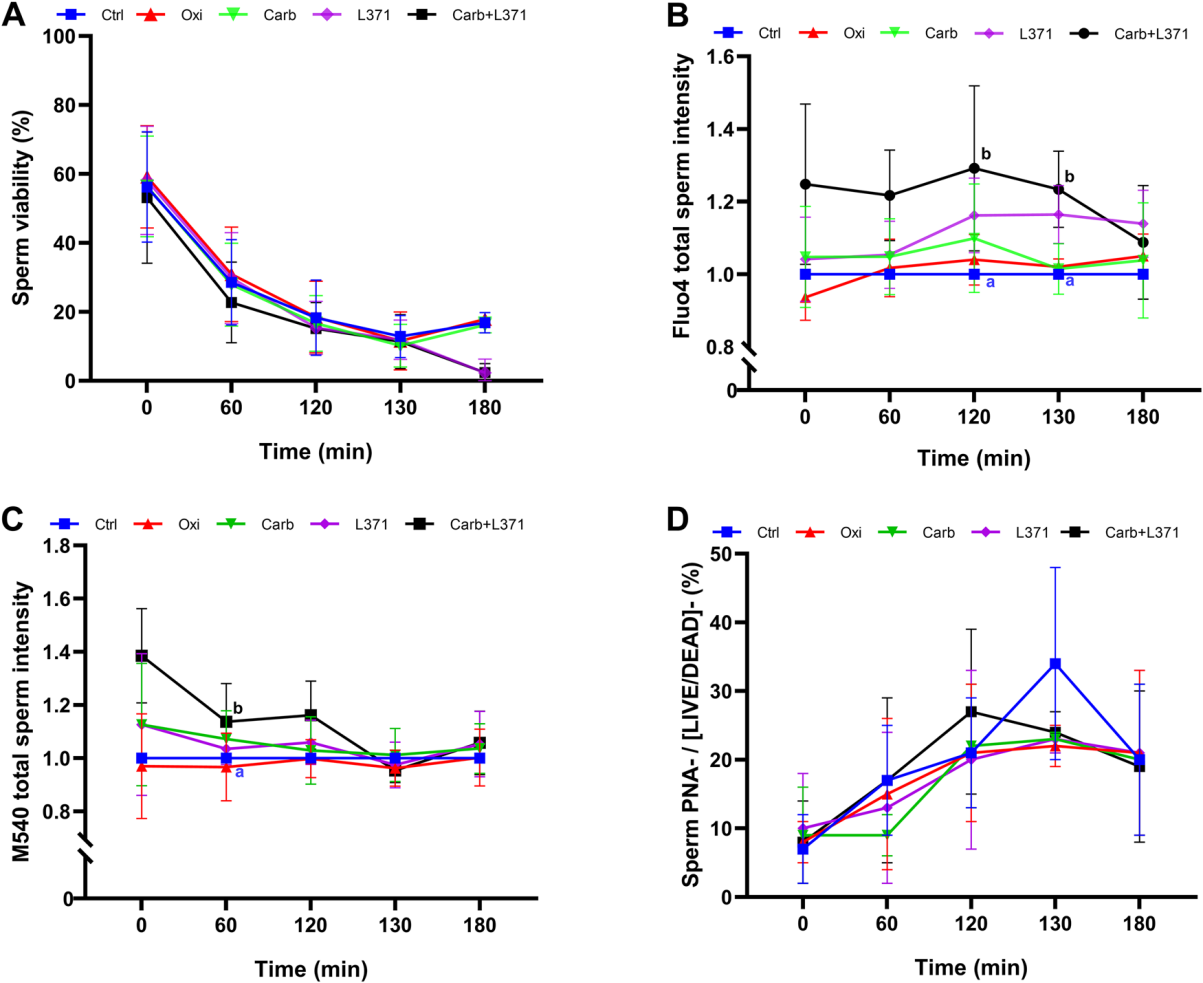
Effect of the oxytocin receptor (OR) agonists (oxytocin and carbotecin), antagonist (L‐371,257) and their combination (carbotecin + L‐371,257) on viability, intracellular calcium, membrane lipid disorder, and acrosomal integrity during in vitro sperm capacitation. (A) Plasma membrane integrity (% viable spermatozoa; SYBR‐14^+^/PI^−^); (B) ratio of Fluo4^+^ intensity in total spermatozoa (viable and non‐viable) normalized to the control. (C) ratio of M540^+^ intensity in total spermatozoa (viable and non‐viable) normalized to the control; (D) percentage of viable spermatozoa with an exocytosed acrosome (PNA^−^/[Live/Dead]^−^). Results are expressed as the mean ± SD (*N* = 5). To simplify the plot, the treatment with oxytocin + L‐371,257 is not represented, as their values were similar to those of the control.

Incubation of spermatozoa with L‐371,257 + Carb resulted in an increase of intracellular Ca^2+^ levels (Fluo4‐AM staining) compared with the control (*p *< 0.05) at 120 min, and after adding progesterone (i.e., at 130 min; Figure 5B; non‐normalized data are shown in Supporting Information File 1). Neither the single addition of either L‐371,257 or Carb, nor the other treatments with oxytocin or oxytocin + L‐371,257 led to that increase. Similar to the findings for intracellular Ca^2+^ levels, only the treatment with Carb + L371,257 showed a significant increase in membrane lipid disorder (M540 intensity) at 60 min, compared with the control (Figure 5C; non‐normalized data are shown in Supporting Information File 1).

Activating or blocking the OR with agonists or antagonists did not impair sperm viability (SYBR‐14/PI) at any of the evaluated time points (Figure 5A). Regarding acrosomal integrity (PNA^+^/[LIVE/DEAD]^−^), no significant differences between treatments were observed at any of the time points evaluated (Figure 5D).

### 3.6 The Oxytocin Receptor Is Not Involved in the Regulation of Mitochondrial Activity or Oxidative Balance

**FIGURE 6:**
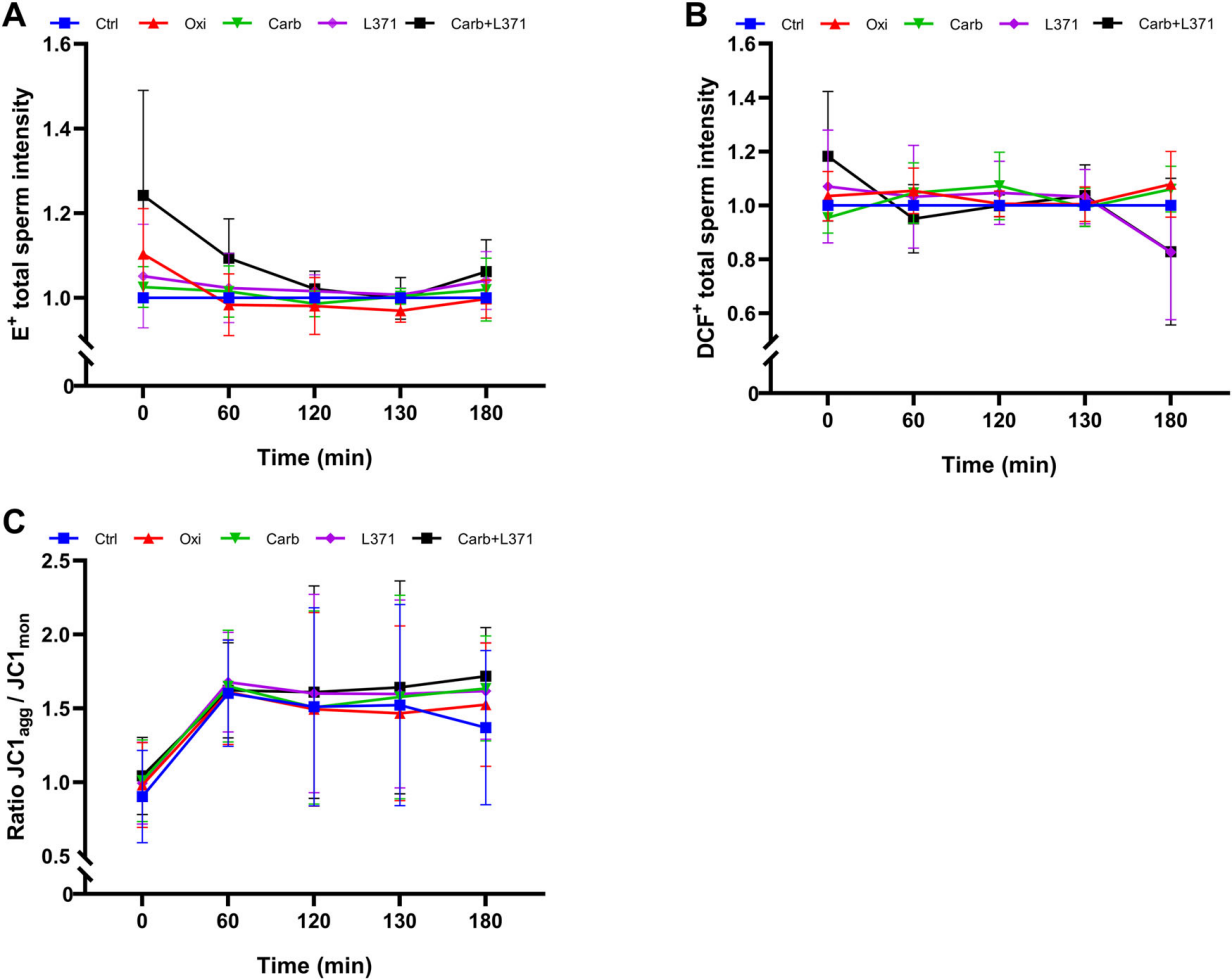
Effect of the oxytocin receptor (OR) agonists (oxytocin and carbotecin), antagonist (L‐371,257), and their combination (carbotecin + L‐371,257) on intracellular superoxide levels, total ROS levels and mitochondrial activity during in vitro sperm capacitation. (A) Ratio of E^+^ intensity in total spermatozoa (viable and non‐viable) normalized to the control; (B) ratio of DCF^+^ intensity in total spermatozoa (viable and non‐viable) normalized to the control; (C) ratio of intensity between JC‐1_agg_ and JC‐1_mon_ in viable spermatozoa. Results are expressed as the mean ± SD (*N* = 5). To simplify the plot, the treatment with oxytocin + L‐371,257 is not represented, as their values were similar to those of the control.

Regarding intracellular superoxide levels (HE/Yo‐Pro‐1), no significant differences between treatments were observed at any time point during in vitro sperm capacitation (Figure 6A; *p *> 0.05). Similarly, no significant variations were detected between treatments for intracellular ROS levels (H_2_DCFDA/PI; Figure 6B; *p *> 0.05). Finally, the activation or blockage of the OR did not affect MMP (*p *> 0.05), as evaluated by the JC‐1_agg_/JC‐1_mon_ ratio, during in vitro sperm capacitation (Figure 6C).

## 4 DISCUSSION

In this study, we investigated the origin and presence of the OR in mammalian spermatozoa, using the pig as a model, and we addressed its potential role during in vitro capacitation. While germ cells, including spermatids, were found to express the OR—which would lead one to advise that it is incorporated into spermatozoa during spermiogenesis—, the presence of this protein in the epididymal apical blebs could suggest that there is also incorporation of the receptor during epididymal maturation. On the other hand, we found that the activation of the OR during sperm capacitation increased intracellular Ca^2+^ levels and membrane lipid disorder—two known capacitation markers—, without affecting sperm viability or acrosome integrity.

First, the presence of the OR in spermatozoa was confirmed by immunoblotting, as two bands of approximately 41 and 55 kDa were identified in blots. These two bands would correspond to the non‐glycosylated and glycosylated forms of the receptor, as the sequence of the polypeptide chain indicates that the OR has three glycosylation sites (Uniprot entry name: P32306). To our knowledge, immunoblotting of the OR has not been performed in spermatozoa, but it has been conducted in the male reproductive organs. Interestingly, the band corresponding to the OR was also of 55 kDa when testicular extracts of humans, macaques, and horses were examined.^47^, ^48^ Furthermore, a study involving guinea pig myometrial cells revealed that digesting the OR with F‐endoglycosidase resulted in a single band of approximately 40 kDa,^49^ thus supporting that the two bands observed in our blots corresponded to non‐glycosylated (40 kDa) and glycosylated (55 kDa) forms.

Regarding localization in the sperm cell, the OR was identified in the equatorial segment, post‐acrosomal region, connecting piece (neck), and midpiece. Similar to our results, the only study that has described the presence of the OR in human spermatozoa found it in the posterior area of the head, connecting piece (neck), and midpiece.^26^ The localization pattern would be related to the signaling pathway triggered by the activation of the OR, which is a GPCR. Related to this, it is worth mentioning that different types of G‐protein subunits have been identified and localized in mammalian spermatozoa. Specifically, in human spermatozoa, the Gα_q/11_ subunit was observed in the equatorial segment and acrosome; the Gα_i2_ subunit in the midpiece and acrosome; and the Gα_i3_ subunit in the post‐nuclear region, midpiece, and principal piece.^50^ In mouse spermatozoa, the Gα_q/11_ subunit was localized in the acrosome, connecting piece, and flagellum; the Gα_i2_ subunit in the acrosome, midpiece (weak), and principal piece; the Gα_i3_ subunit in the equatorial segment and principal piece; and the Gα_o_ subunit in the acrosome, connecting, and principal piece.^51^ The specific expression of G‐protein subunits in mammalian spermatozoa, together with data from the present work, suggests that the OR could be spatially and temporally involved in sperm capacitation via the modulation of PKA through inhibition of mACY and/or phospholipase C‐mediated pathways. This was investigated further and will be discussed later.

As far as the localization of the OR in the testis and epididymis is concerned, IHC analyses (i.e., bright‐field microscopy) indicated that the receptor was present in the germ cells of the seminiferous epithelium, with strong labeling in spermatids and residual bodies; yet, no immunostaining was observed in testicular spermatozoa, cytoplasmic droplets, or myoid cells. In addition, the cytoplasm and membrane of both Sertoli and Leydig cells showed positive immunolabeling for the OR. Our results are consistent with previous studies, as the OR has been identified in the spermatogonia, spermatids, and cytoplasmic droplets of horses,^48^ as well as in germ cells and acrosomal vesicles of spermatids in sheep.^52^ Regarding Sertoli and Leydig cells, this receptor has been found to be expressed in different mammalian species (humans;^47^ marmoset monkey;^53^ sheep;^52^ and horses, but in this case only in Leydig cells ^48^). Like what was observed herein for pigs, the myoid cells in humans and sheep are devoid of the OR,^47^, ^52^ which contrasts with the case of horses, where myoid cells express the receptor.^48^ To the best of our knowledge, no study has previously reported the expression of the OR in testicular spermatozoa, as we do in the current work using confocal microscopy. While the function of the OR during spermatogenesis is not clearly understood, it has been suggested to play a role during the spermiation mediated by Sertoli cells in mouse,^54^ rat,^55^, ^56^ and sheep,^52^ and in the proliferation of spermatogonia in the pre‐pubertal mouse.^57^ On the other hand, the presence of the OR in Leydig cells could be explained by the function of this hormone, which regulates the expression of the enzymes involved in the synthesis of testosterone and its conversion into derivatives, such as dihydrotestosterone.^52^, ^57^, ^58^, ^59^ As far as myoid cells are concerned, the role of the OR in these cells has not been elucidated, notwithstanding this receptor has been suggested to be involved in tubular contraction and sperm transport toward the rete testis.^60^

Regarding the epididymis, the OR was found to be localized in the supranuclear region of principal cells, in the apical blebs and their vesicular derivatives within the lumen, as well as in apical, some basal cells, and smooth muscle cells of the epididymal duct. This concurred with previous studies where the OR was identified in the principal cells of the epididymis in humans,^61^ other primates,^59^ horses,^48^ and sheep.^52^ Remarkably, this is the first time that the OR has been detected in the apical blebs and their derivatives in the epididymal lumen originating from the epididymal epithelium. The expression of the OR in the smooth muscle cells of the epididymal wall was also observed in other mammalian species.^52^, ^53^, ^59^ In fact, the function of the OR in the epididymis has traditionally been associated with the contractility of the muscle wall and the transport of spermatozoa throughout this organ.^61^, ^62^ The function of the OR in the epithelial cells of the epididymis, however, remains unknown. It has been suggested that the OR may modulate the activity of 5α‐reductase, which is expressed by epididymal epithelial cells, thereby influencing the concentration of dihydrotestosterone, a hormone upon which the epididymis depends^18^, ^52^ As far as apical blebs are concerned, they are structures that originate from the principal cells through apocrine secretion. It is known that these structures contain EVs, known as epididymosomes, which are released when apical blebs disaggregate, thereby delivering their contents into the epididymal lumen,^63^, ^64^, ^65^ as we have also observed in our ultrastructural description, which confirms previous studies.^35^ These released epididymosomes can bind to other cells of the epididymal epithelium, exerting a paracrine effect, or bind to spermatozoa during their maturation process in the epididymis.^63^, ^64^ In this context, our electron microscopy observations raise the question of which specific vesicular substructure within the apical blebs contains the OR. Although this study cannot answer that question, it highlights the need for a whole classification and systematic characterization of apical blebs and their derivatives in the epididymis. In the present work, we observed, through both bright‐field and confocal microscopy, that the OR is present in the apical blebs, suggesting that the epididymosomes inside these structures could carry the OR. Remarkably, the triple immunofluorescence protocol involving CD81, caveolin‐1, and the OR showed that these three proteins colocalized in these apical blebs; it is worth mentioning that both CD81 and caveolin‐1 are markers of EVs.^46^ These observations, together with the fact that confocal microscopy showed that the OR is only expressed in the post‐acrosomal region of testicular spermatozoa, whereas spermatozoa recovered from the epididymal have the OR in both the post‐acrosomal region and the tail, suggest that apical blebs/epididymosomes may be an alternative source of this receptor during epididymal maturation. Consistent with these observations, the OR was found in the equatorial segment, post‐acrosomal region, connecting piece, and midpiece of ejaculated spermatozoa. This suggests that not only the EVs released by the epididymis but also those produced by other cells of the male genital tract could be involved in the incorporation of this receptor into spermatozoa.^63^, ^65^ Another explanation for the different localization of the OR between epididymal and ejaculated spermatozoa could be the redistribution of the receptor upon ejaculation, or discrepancies between immunocytochemistry and immunofluorescence protocols. Further research is needed to clarify this issue.

In the present study, we also examined the role of the OR during in vitro capacitation and acrosome reaction using agonists, antagonists, and combinations of both. The concentrations of the agonists were similar to the physiological levels reported in the literature.^25^, ^37^, ^38^ Regarding the analyzed cytometry parameters, no significant differences between treatments were observed, except for the increase in intracellular Ca^2+^ levels and membrane lipid disorder. In this study, the agonist plus antagonist treatment (Carb + L‐371,257, but not Oxy + L‐371,257 [not represented]) resulted in an increase in membrane lipid disorder at 60 min, as well as the intracellular Ca^2+^ levels after 120 and 130 min of in vitro capacitation. Recently, a study using cell lines and primary cultures of endometrial cells observed that treatment with antagonist L‐371,257 increased the amount of the OR on the cell surface and enhanced the endogenous oxytocin response via the G_q_‐phospholipase C‐inositol triphosphate pathway, suggesting that L‐371,257 acts as a pharmacochaperone.^66^ Pharmacochaperones are defined as molecules that stabilize protein binding by acting as either an agonist, antagonist, or allosteric modulator.^67^, ^68^ The explanation for the effects of L‐371,257 + Carb on spermatozoa may stem from their roles as a pharmacochaperone and agonist, respectively, which would result in an increase in membrane lipid disorder and intracellular Ca^2+^ levels. It is reasonable to ask why the treatment with Oxy + L‐371,257 had no effect on sperm physiology, whereas the Carb + L‐371,257 one did. A possible explanation would be the shorter half‐life of oxytocin compared with carbetocin.^69^ Another important aspect is that the OR has states of high or low affinity, modulated by cholesterol and divalent cations, which induce conformational changes in the OR and facilitate agonist binding.^70^, ^71^ Membrane lipid disorder is an early event in capacitation, triggered by sperm exposure to bicarbonate, which activates phospholipid scramblases that reorganize the lipid bilayer, facilitating the redistribution of membrane cholesterol and its subsequent efflux through acceptor proteins, such as albumin.^32^, ^72^ These circumstances could explain why the treatment with Carb + L‐371,257 was the only one that affected intracellular Ca^2+^ and membrane lipid disorder. On the one hand, L‐371,257 would function as a pharmacochaperone by stabilizing the OR; on the other, carbotecin, because of its longer half‐life, would be in equilibrium competing to bind to the OR.^66^

With respect to the intracellular signaling pathway triggered by the OR, the increase in intracellular calcium suggests that, in spermatozoa, this receptor is coupled to a Gα_q/11_ effector protein rather than to a Gα_i_ or Gα_o_. In fact, it has been observed that carbotecin is a selective functional agonist of the OR coupled to Gα_q/11_ proteins.^73^ The activation of the OR could thus stimulate phospholipase C (β1) in spermatozoa, which would generate IP_3_ and DAG from PIP_2_. This IP_3_ would bind to its receptor (IP_3_R), causing the release of Ca^2+^ from cellular stores (acrosome, and mitochondria, although the latter remains controversial ^74^). Additionally, it has been reported that OR activation causes membrane depolarization, which opens voltage‐gated Ca^2+^ channels and allows Ca^2+^ influx. This Ca^2+^ would then bind to calmodulin, stimulating calmodulin‐dependent kinase (CaMK), which would in turn phosphorylate other proteins.^75^ Along with a rise in intracellular Ca^2+^ levels, one would expect an increase in the occurrence of the acrosome reaction, but this was not observed. This could be because the influx of intracellular Ca^2+^ was not sufficient to trigger the acrosome reaction.

Concerning the relevance of the OR in the regulation of sperm motility during in vitro capacitation, no significant differences between treatments were observed. It could be that the whole effect of the OR on sperm motility was not detected because most intracellular signaling of GPCRs is rapid (3–10 min), followed by desensitization and internalization of the OR via β‐arrestin,^5^, ^76^ and sperm motility was assessed later. For this reason, a limitation of this work is that, in order to block the activity of the OR with the antagonist L‐371,257 and to standardize the treatment with the different agonists, a pre‐incubation with each treatment (agonists and antagonists) was performed, resulting in a delay of 30 min before the start of time 0.

## 5 CONCLUSIONS

This study investigated, for the first time, the presence and localization of the OR in testicular and epididymal spermatozoa, as well as its involvement in in vitro capacitation and acrosome reaction using agonists at physiological concentrations. The results observed in this work indicate that the OR originates from spermiogenesis, although other sources of the OR, such as the epididymis through EVs, may be involved in the addition and/or relocalization of the OR in spermatozoa. Regarding the involvement of the OR in sperm physiology, the OR has a modest effect on in vitro capacitation, with an observed impact on intracellular Ca^2+^ and membrane lipid disorder, suggesting that this receptor may act via a G_αq/11_‐PLCβ pathway, and the OR is active at physiological concentrations. Further research interrogating whether modulating the OR affects the ability of spermatozoa to fertilize the oocyte is warranted.

## AUTHOR CONTRIBUTIONS

Jesús Martínez‐Hernández designed and performed the experiments, analyzed the data, and wrote the original draft; Ferran Garriga, Adeel Ahmad, Lorena Padilla, Carolina Maside, and Sergi Bonet performed experiments; Isabel Barranco, Jordi Roca, and Luis Miguel Pastor reviewed the manuscript; and Marc Yeste performed the statistical analysis, reviewed, and edited the manuscript. All the authors read and approved the final manuscript.

## CONFLICTS OF INTEREST STATEMENT

The authors declare no conflicts of interest.

## ETHICS STATEMENT

The authors have nothing to report.

## CONSENT

All the authors are aware of their work and approve of the content of the article and the fact that they are listed as authors of the article.

## Supporting information



Supporting Information

Supporting Information

Supporting Information

## Data Availability

Data will be made available from the authors upon reasonable request.
